# Is there an optimal time to administer postoperative stereotactic radiosurgery in patients with brain metastases? A systematic review of the literature and meta‐analysis

**DOI:** 10.1002/pro6.1214

**Published:** 2023-12-03

**Authors:** Anthony Nwankwo, Danielle D. Dang, Kevin Choe, Samir Kanani, Adam L. Cohen, Mateo Ziu

**Affiliations:** ^1^ University of Virginia School of Medicine – Inova Fairfax Campus Falls Church Virginia USA; ^2^ Department of Neurosurgery Inova Fairfax Medical Campus Falls Church Virginia USA; ^3^ Department of Radiation Oncology Inova Schar Cancer Institute Inova Health System Fairfax Virginia USA; ^4^ Division of Neuro‐Oncology Inova Schar Cancer Institute Inova Health System Fairfax Virginia USA

**Keywords:** metastatic brain cancer, intracranial metastases, stereotactic radiosurgery, adjuvant radiation

## Abstract

Postoperative stereotactic radiosurgery improves local tumor control in patients with metastatic brain cancer. However, the influence of timing on its therapeutic efficacy is unclear. In this study, we performed a meta‐analysis and systematic literature review examining publications that reported the timing of postoperative stereotactic radiosurgery (SRS) for patients with intracranial metastases. Our primary outcomes included median overall survival and rates of local and regional failure, while secondary outcomes examined the incidence of treatment‐related adverse events. Correlations between median SRS timing and these variables were assessed using linear regression and publication bias was appraised via Egger's test. Our study resulted in 22 articles comprising 1338 patients. The median timing of adjuvant SRS spanned 14.5 to 41 days. There was a significant negative study‐level correlation of median time to SRS with regional failure (*p* = 0.043, R^2^ = 0.32) but not with overall survival (*p* = 0.54, R^2^ = 0.03) or local failure (*p* = 0.16, R^2^ = 0.14). Additionally, there was significant heterogeneity within the reports (*p*<0.0001). In conclusion, our analysis demonstrated that postoperative SRS timing did not influence local failure rates which may in part be due to significant variability between individual study designs and patient demographics. Further research is warranted to elucidate the role of timing for postoperative SRS on oncologic outcomes.

## Graphical abstract

Postoperative stereotactic radiosurgery improves local tumor control in patients with metastatic brain cancer. However, the influence of timing on its therapeutic efficacy is unclear. In this study, we performed a meta‐analysis and systematic literature review examining publications that reported the timing of postoperative SRS for patients with intracranial metastases. Our primary outcomes included median overall survival and rates of local and regional failure, while secondary outcomes examined the incidence of treatment‐related adverse events. Correlations between median SRS timing and these variables were assessed using linear regression and publication bias was appraised via Egger's test. Our study resulted in 22 articles comprising 1338 patients. The median timing of adjuvant SRS spanned 14.5 to 41 days. There was a significant negative study‐level correlation of median time to SRS with regional failure (*p*=0.043, R^2^=0.32) but not with overall survival (*p*=0.54, R^2^=0.03) or local failure (*p*=0.16, R^2^= 0.14). Additionally, there was significant heterogeneity within the reports (*p*<0.0001). In conclusion, our analysis demonstrated that postoperative SRS timing did not influence local failure rates which may in part be due to significant variability between individual study designs and patient demographics. Further research is warranted to elucidate the role of timing for postoperative SRS on oncologic outcomes.

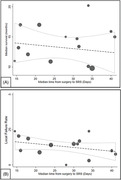

## 1 INTRODUCTION

Metastatic brain cancer (MBC) is the most common form of intracranial malignancy.^1^ MBC affects 20 to 40% of metastatic cancer patients and is associated with poor prognosis despite the advent of various adjuvant therapies.^2^ Current treatment options include whole‐brain radiotherapy (WBRT), surgical resection, stereotactic radiosurgery (SRS), and systemic therapy. These modalities can be amalgamated in different combinations and demonstrate varying efficacy based on patient‐specific factors such as treatment goals, intracranial tumor burden, and the molecular characteristics of the primary neoplasm.^3^, ^4^

WBRT was once considered the gold standard for treating MBC after complete resection, offering symptom relief, improved local tumor control, and prevention of central nervous system (CNS) recurrence.^5^, ^6^, ^7^ However, irreversible cognitive decline as a sequela of WBRT necessitated the development of additional radiation techniques such as SRS, a non‐invasive ablative technique using a three‐dimensional coordinate system to improve the precision of radiation delivery and minimize neurotoxicity.^8^, ^9^ The role of SRS in MBC treatment is well‐established in the current literature supported by evidence of improved neurocognitive function and equivalent 1‐year local control rates compared to postoperative WBRT.^1^, ^10^, ^11^, ^12^

While SRS dosimetric parameters continue to be refined, the impact of its timing on therapeutic efficacy and patient outcomes remains undetermined. It has been hypothesized that hypoxia within the tumor microenvironment may attenuate the lethality of high‐dose radiation and decrease its anticancer efficacy when employed closer to the time of surgery.^13^ On the other hand, increased time from surgery to SRS during which viable malignant cells could begin to proliferate and spread could contribute to local and regional failure. In practice, SRS timing ultimately varies based on numerous clinical, pathophysiologic, and socioeconomic factors, thereby leading to a lack of consensus among clinicians and rendering optimal radiosurgery timing unknown.^14^, ^15^

We present a systematic review and meta‐analysis of the literature evaluating the relationship of time to SRS after neurosurgical resection of MBC to delineate potential effects on oncologic outcomes and to further explore the variety of clinical and pathophysiologic factors which may contribute to adjuvant treatment efficacy.

## 2 MATERIALS AND METHODS

### 2.1 Study selection

A systematic literature review was performed in December 2022 in accordance with the Preferred Reporting Items for Systematic Reviews and Meta‐Analyses (PRISMA) guidelines, and included a database search of PubMed, Cochrane Library, Excerpta Medica DataBASE, and OVID.^16^ Search terms included: (brain neoplasms OR brain tumor OR brain cancer) AND (metastasis or neoplasm metastasis) AND (radiosurgery OR gamma knife OR Linac). All human studies restricted to the English language were included. Two authors independently screened and reviewed the titles, abstracts, and manuscripts, with the bibliographies of each manuscript reviewed for inclusion of additional articles, and this process was iteratively performed until completion. The meta‐analysis included studies with a sample size of at least 20 patients with histopathology‐proven metastatic brain cancer who underwent neurosurgical resection of at least one metastasis followed by SRS. Exclusion criteria included: inaccessible full text articles, insufficient information regarding radiosurgery timing and/or survival, primary CNS histopathology, and location of metastases outside of the intracranial compartment.

### 2.2 Data extraction

The following data points were extracted for analysis: study sample size, median age (years), sex (% female), disease severity, extent of neurosurgical resection, location of treated intracranial metastases, single brain metastasis (%), primary breast cancer (%), total SRS dose (Gy), postoperative timing of SRS administration (days), treatment cavity volume (mL), treatment complications including surgical cavity radionecrosis, the incidence of local and regional failure, and median overall survival (OS, months). Local failure was defined as metastatic recurrence at the surgical site whereas regional failure was defined as the recurrence of intracranial metastasis at a site untreated with surgery and SRS. Recursive Partitioning Analysis class (RPA), Graded Prognostic Assessment (GPA), and Medical Research Council (MRC) Scale scores were used to delineate disease severity.^17^, ^18^, ^19^

### 2.3 Statistical analysis

All statistical analyses were performed using Stata v17 software. Because individual patient data from the studies were not available, meta‐regression was used to quantify the relationship between time to postoperative SRS and outcomes at a study level. Linear regression weighted based on sample size was performed using a random effect model for median OS, percent local failure, percent regional failure, and median time to postoperative SRS. Regression was first performed using univariate regression. The effect of covariates on the relationship between median time to SRS and outcomes was assessed using multivariate regression weighted based on sample size using a random effect model using median time to SRS and each covariate individually in turn as independent variables. For heterogeneity and publication bias analyses, standard error of the median was estimated using the median divided by the square root of the sample size. Publication bias was evaluated via Egger's test analysis in conjunction with a visual inspection of the funnel plots. For all analyses, a p‐value of less than 0.05 was used as a cut‐off for statistical significance.^20^

## 3 RESULTS

### 3.1 Systematic literature review

**FIGURE 1:**
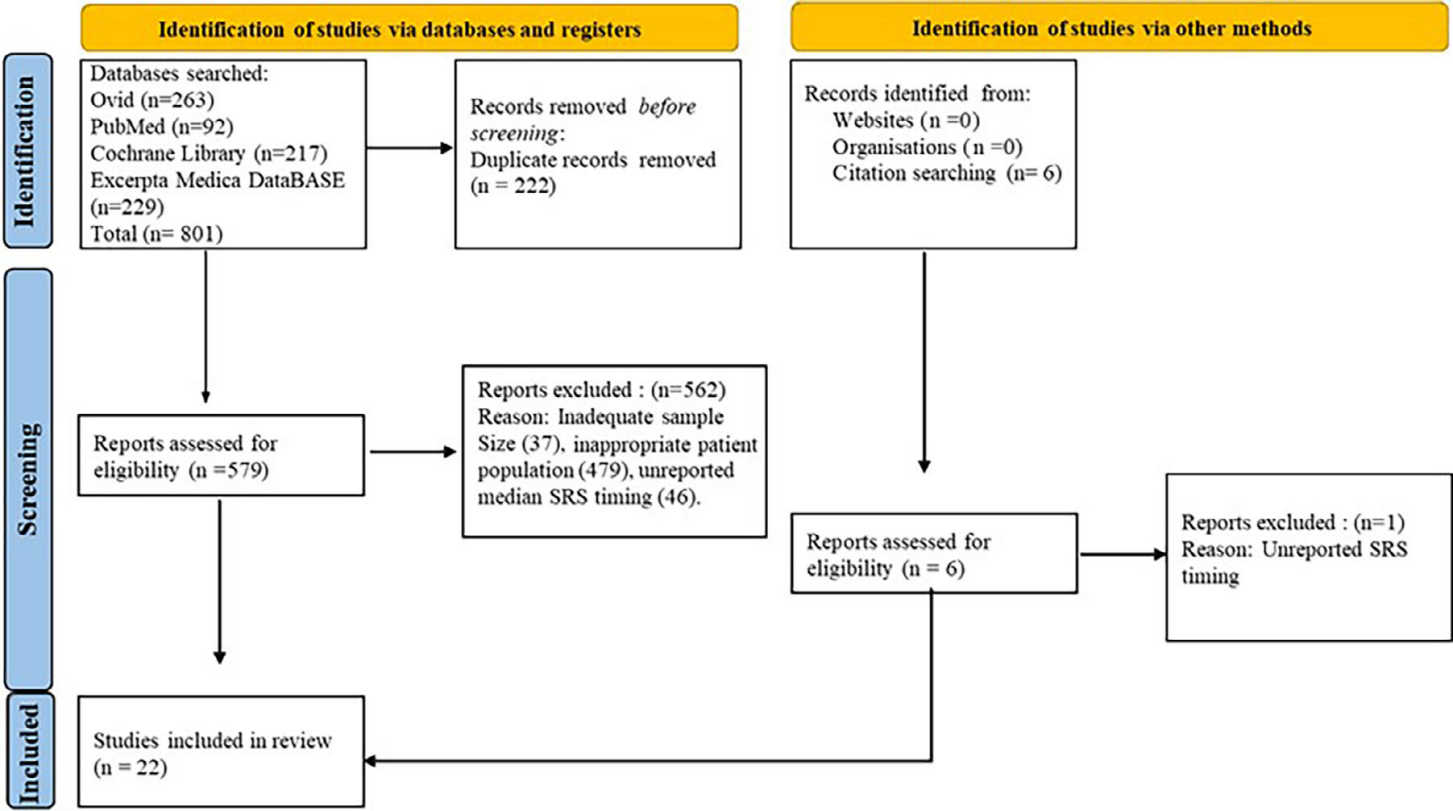
PRISMA flowchart used to critically examine the impact of timing of adjuvant SRS on patients with metastatic brain cancer.

A total of 579 studies were screened from which 22 articles published between 2008 to 2022 met inclusion criteria for analysis (Figure 1). Studies were primarily excluded for inadequate sample size, inappropriate patient population, or unreported SRS timing. Aggregate values for each study variable are reported in Table 1 while individual data for every included article is listed in Suppl. Figure S1.

**TABLE 1:** The minimum, maximum, and average values for each study variable extracted from the included articles as well as the total number of articles that reliably reported each value.

Study Variable	Minimum Value	Maximum Value	Mean Value	Number of Reports (%)
Patient sample size	21	176	109	22 (100%)
Median age (years)	55	67	61	18 (81.8%)
Female sex (%)	38	76	57	19 (86.4%)
Low disease severity (%)	16.6	69	42.8	8 (36.4%)
Breast cancer primary neoplasm (%)	3.5	36	16.3	21 (95.4%)
Single intracranial metastasis (%)	0	100	68.1	17 (77.3%)
Infratentorial lesions treated (%)	8	33.3	20.7	9 (40.9%)
Gross total resection (%)	68	100	84	16 (72.7%)
Median time to SRS (days)	14.5	41	27.8	17 (77.3%)
Median SRS dose (Gy)	9	30	19.5	14 (63.6%)
Median treatment cavity volume (mL)	3.9	27.2	15.5	14 (63.6%)
Median overall survival (months)	10	20.2	15.1	18 (81.8%)
Local failure rate (%)	0	26	13	20 (90.9%)
Regional failure rate (%)	21	72	46.5	16 (72.7%)
Surgical cavity radionecrosis (%)	0	27	13.5	18 (81.8%)

### 3.2 Patient demographics

A totle of 1338 patients were analyzed. The median age ranged from 55 years to 67 years (SD = 2.9). The overall sex distribution was calculated by multiplying the percent female for each study by the total number of patients in each study, summing those numbers, and then dividing by the total number in all the studies. Sex distribution comprised a female‐to‐male ratio of 1:0.85 with the female population ranging from 38% to 76% across studies. Female sex did not significantly correlate with the percent of breast cancer in the series (R^2^ = 0.09, *p* = 0.23).

### 3.3 Tumor characteristics

The percent of patients in each series with low disease severity, denoted by an RPA class I, GPA 1–2, or an MRC scale of 1, ranged from 16.6% to 69.0%. The percentage of patients with breast cancer ranged from 3.5% to 36.0% (mean 16.3%, SD = 7.7). Treatment of single metastatic lesions varied widely, yet comprised 68.1% of the analyzed cohort. In contrast, infratentorial metastatic lesions accounted for a minority of the population with a range of 8.0% to 33.0%. Gross total resection (GTR) was reportedly achieved in 68.0% to 100.0% of patients.

### 3.4 Radiotherapy treatment regimen

The median time to postoperative SRS, defined as the time in days from surgical resection of the metastatic tumor to the administration of SRS to the surgical cavity, ranged from 14.5 days to 41.0 days with a mean of 27.8 days (SD = 9.1). The median total SRS dose ranged from 9 to 30 Gy (SD = 4.4) in which three‐fourths of the series had a median dose between 15–19 Gy. The corresponding median treatment cavity volume ranged from 3.9 mL to 27.2 mL with a mean of 15.5 mL (SD = 5.9).

### 3.5 Patient outcomes

The median OS for patients ranged from 10 months to 20.2 months (SD = 3.2), which is similar to what has previously been reported for this population group.^10^ The incidence of regional failure ranged from 21.0% to 72.0% of the patient population. Surgical cavity radionecrosis was a relatively uncommon finding with nine studies noting no occurrence; however, one study did report an incidence as high as 27.0% in their cohort (SD = 7.7). Other complications related to SRS or surgery were only documented in seven articles and included symptomatic cerebral edema, culture‐negative meningitis, and intracranial hemorrhage (Suppl. Figure S2). Otherwise, no wound‐related complications were reported.

### 3.6 Meta‐regression

Median timing to SRS did not significantly correlate with percent females, percent GTR, or percent infratentorial lesions. There was a borderline correlation between average age and time to SRS, with older patients undergoing SRS at increased time intervals after surgery (R^2^ = 0.2, *p* = 0.07). A similar correlation was found between SRS timing and percent of patients with low disease severity, in which patient cohorts with perceived lower risk underwent SRS treatment at a comparatively delayed time interval (R^2^ = 0.7, *p* = 0.08).

**FIGURE 2:**
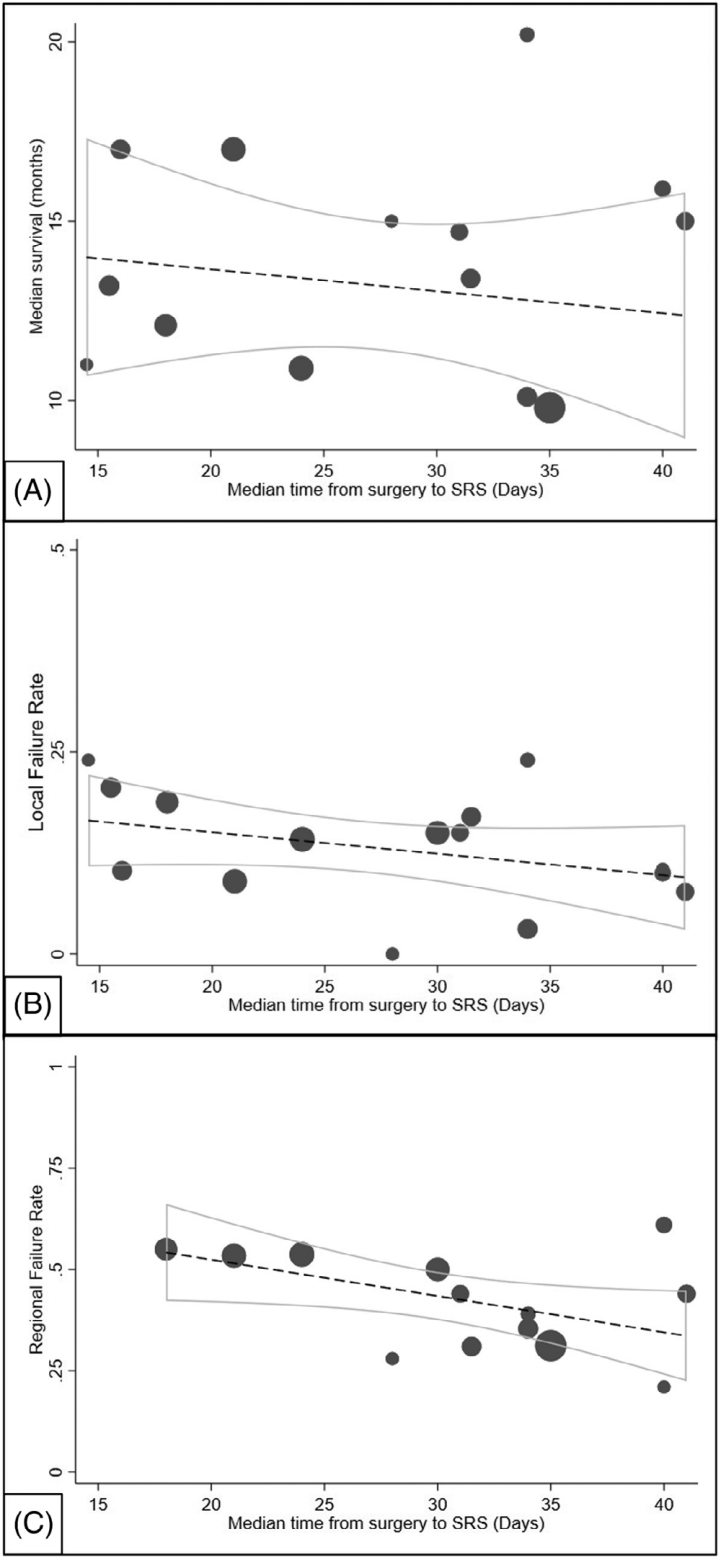
Meta‐regression plot of median time to postoperative SRS and (A) median overall survival, (B) local failure rate, and (C) regional failure rate. In each graph, the size of the circle is proportional to the sample size of the article. The dashed line represents the meta‐regression line, and the solid curves are the 95% confidence intervals.

Linear regression yielded a significant negative study‐level correlation between median time to SRS and percent regional failure (*p* = 0.04, R^2^ = 0.3). After inclusion of female percentage in the meta‐regression model, the significance became borderline (*p* = 0.05), while the effect of female sex percentage on regional failure rate remained significant (*p* = 0.01). Additional variables tested, including percent of breast cancer, infratentorial disease, percent GTR, percent low disease severity, percent single intracranial metastasis, and median age, did not impact the relationship between median time to SRS and regional failure rate. There was no association between median time to SRS and median OS (*p* = 0.5, R^2^ = 0.03) nor percent local failure (*p* = 0.2, R^2^ = 0.1) (Figure 2).

### 3.7 Study heterogeneity and bias

Significant study heterogeneity for estimating the median time to SRS using a random effects model was evident (*p*<0.0001). Other measures examined, such as the ratio of median overall survival, local recurrence rate, or regional recurrence rate to median time to SRS showed similar heterogeneity (*p*<0.0001)(Suppl Figure S3). Heterogeneity remained after removing individual studies. Significant publication bias for articles included in the meta‐analysis was present for the estimate of median time to SRS (Egger's test, beta = 4.22, SE of beta = 0.929, *p*<0.0001), with possible missing smaller studies with smaller median time to SRS (Suppl Figure S4)

## 4 DISCUSSION

With the rise of postoperative SRS use in the treatment of MBC, optimization of its workflow may maximize its known benefit to neurologic function and local tumor control.^1^, ^10^, ^11^, ^12^, ^14^, ^21^, ^22^ Studies have attempted to explore the impact of SRS timing after neurosurgical resection on clinical outcomes; however, results are mixed.^10^, ^23^, ^24^ In this study, we conducted a systematic review and meta‐analysis of the literature to evaluate the relationship between postoperative SRS timing and oncologic outcomes in MBC patients.

The median postoperative SRS timing in our analysis ranged from two to six weeks.^14^, ^25^ Variables influencing the timing for postoperative SRS are multifactorial including socioeconomic, clinical, and logistical components. Across the articles included in our study, a longer time from surgery to SRS seemed to be associated with older age and lower‐risk status cohorts. A possible explanation for these findings could be increased surgical recovery times in older patients, resulting in a delayed evaluation for adjuvant radiotherapy and oncological outpatient follow‐up.^26^ Furthermore, studies with a higher percentage of low‐risk patients may represent cohorts with a favorable prognosis, which may decrease the urgency for immediate initiation of radiosurgery.^27^ Lastly, it is possible that institutions whose populations are older and lower risk may have other systemic reasons for doing SRS later. Relevant socioeconomic and logistical variables were not analyzed in relation to SRS timing in the included articles. Similarly, clinical variables like the incidence of complications related to neurosurgical resection and length of stay (LOS) were scarcely mentioned. Neurosurgical complications, which impact LOS and a patient's ability to undergo adjuvant therapy were documented in only three of the 22 articles evaluated. Of note, no articles reported incidence of wound dehiscence or surgical site infection. Since the majority of studies did not comment on complication incidence, its impact on the timing of postoperative SRS cannot be assessed (Suppl. Figure S2).^14^, ^28^ Finally, one group described the exclusion of a patient from their study as a result of missing the 30‐day SRS window due to surgical complications, also highlighting a need to understand and include patients who never make it to SRS.^10^, ^14^, ^29^

Additional factors yielding delays in postoperative SRS, such as access to transportation, have been previously discussed in the literature but not systematically studied in terms of oncologic outcomes.^30^ A study by O'Brien et al. found that logistical factors accounted for 33% of the delays in SRS treatment, while management and treatment of systemic disease and comorbidities accounted for 22.6% and 13.0% of the delays, respectively.^15^ Within their cohort, 12.6% of patients never received SRS despite standardized referral practices. Similarly, another group noted that rapid CNS progression, uncontrolled systemic disease requiring prolonged hospitalization, and loss of follow‐up resulted in 20% of their cohort not receiving adjuvant SRS therapy.^31^ Future articles should include these variables and document patients who do not have SRS to better understand how they impact healthcare processes, delivery of treatment, and survival outcomes.

Our analysis did not show a significant relationship between SRS timing and median OS. Patient survival is mediated through a variety of oncologic variables including but not limited to primary cancer histology, extracranial disease status, local and regional failure, and treatment tolerance. Indeed, postoperative radiation has not been shown to improve survival, therefore, it is not surprising that time to SRS does not seem to relate to survival at an institutional level.^15^

Our results showed that the median time to postoperative SRS did not significantly correlate with local failure rates, similar to Brennan et al,^31^ yet contrary to findings from other individual cohort studies.^15^ One study suggested that administering SRS within three weeks of surgery was important to minimize the risk of local tumor recurrence independent of variables, such as extent of tumor resection, primary cancer histology, or the size of the surgical cavity to be treated.^24^ Conversely, another study suggested that delaying postoperative SRS for more than 3–4 weeks after the initial resection could maximize spontaneous tumor bed ischemia and reduce the volume of radiation needed for effective treatment.^10^, ^23^ The lack of significance in the meta‐analysis presented herein is likely attributed to the significant level of heterogeneity in study methodology, limited range of reported local control rates, inability to account for length of follow‐up, as well as cohort demographics. Moreover, our meta‐analysis investigated this relationship at an institutional level, in contrast to the analyses performed at an individual level seen in these articles. At present, the temporal relationship between adjuvant SRS timing and local failure remains unclear.

Regarding regional failure rates, this study showed that delayed administration of postoperative SRS was significantly associated with decreased regional failure rates. Selection bias in the form of confounding by the severity of the patient's disease is possible as lower disease severity was associated with a longer time to SRS, however, when adjusting for other markers of severity there were no effects on this relationship. We also cannot exclude other forms of selection bias or institution‐level correlations between resources or care coordination and clinical variables. Although causation cannot be implied from our data, one hypothesis is that providers may prioritize control of extracranial disease when intracranial risk is low while waiting for patients to fully recover from surgery prior to undergoing SRS, though our data cannot test this hypothesis.^24^ The presence of active extracranial disease in the patient cohort was evident in several articles, with some attributing its presence as the reason for delayed SRS administration and others noting its significant negative impact on overall survival.^23^, ^32^, ^33^ Ongoing adequate management of a patient's systemic disease in the immediate postoperative period may be important to lower the risk of developing new CNS metastases and concomitantly benefit existing intracranial malignancy depending on the histopathologic subtype and the systemic treatment being used.^34^, ^35^ Future studies should include information on the systemic therapies used in parallel during this course.

Our study also investigated the potential adverse effects of postoperative SRS to determine if variation in timing also impacted their incidence, particularly radionecrosis. Findings were largely nonexistent with no wound‐related complications described. Likewise, nine studies in total reported no evidence of radionecrosis, conflicting with previous estimates in the literature approaching up to 30%.^36^, ^37^, ^38^, ^39^, ^40^ Potential explanations for these results include limited lengths of follow‐up or the methods used to confirm radionecrosis, including histopathologic analysis via tissue biopsy versus magnetic resonance imaging (MRI). For example, the diagnostic accuracy of radionecrosis is limited by the difficulty in distinguishing it from tumor recurrence using imaging alone since both radionecrosis and tumor recurrence can present as edematous ring‐enhancing lesions.^41^, ^42^ Finally, associations between the incidence of radionecrosis, median time to SRS, radiation dose, and location of the metastatic tumor were not investigated in the studies. Standardizing the diagnostic methods used to confirm and report radionecrosis will in turn improve our understanding of the impact of these various factors, including the timing of adjuvant SRS treatment, on its development.

The limitations of our study stem from its cross‐sectional design and meta‐analysis. Although many potential factors influencing postoperative SRS timing were not formally documented in the evaluated studies, the heterogeneity in their methodology, patient population, and primary outcomes possess a greater barrier to research on this topic (Suppl Figure S3)(Suppl Figure S4). Important variables known to contribute to SRS‐related treatment outcomes, including number of metastases, RPA class, and primary malignancy, differed significantly between the studies.^21^ Additionally, the primary clinical questions and aims of each article differed, leading to significant variations in overall study design.^27^, ^43^, ^44^ Finally, this meta‐analysis evaluated relationships at an institutional level, which may differ from correlations at the individual patient's level. The small number of studies and the heterogeneity amongst them reduces the power of our analyses. Due to significant publication bias, our literature search may not fully encompass the full spectrum of postoperative SRS timing, possibly not accounting for smaller studies with shorter times to SRS. Our analysis supports the need for larger controlled cross‐institutional studies with the ability to accurately account for confounders, identify patterns of treatment‐related adverse effects, stratify patients based on postoperative SRS timing, and comprehensively report the socioeconomic, clinical, and logistical factors that influence timing of SRS administration.

## 5 CONCLUSION

Postoperative SRS is a safe and effective treatment for patients with metastatic brain cancer most often employed between two to six weeks after tumor resection. This meta‐analysis showed no correlation between median postoperative SRS timing and local failure rate and median OS. Our conclusions are limited by significant variability in the study population and publication methodology. Therefore, further research through larger‐scale studies with consistent methodologies is necessary to better understand these potential relationships, optimize radiosurgery workflow, and improve outcomes in this patient population.

## AUTHOR CONTRIBUTIONS

A.N., D.D., K.C., S.K., A.C., and M.Z., contributed to the study's conception and design. Material preparation, data collection, and analysis were performed by A.N., D.D., and A.C. The first draft of the manuscript was written by A.N., D.D., while K.C., S.K., A.C., and M.Z. commented on previous versions of the manuscript. A.N., D.D., K.C., S.K., A.C., and M.Z., read and approved the final manuscript. M.Z. and A.C. supervised the study.

## CONFLICT OF INTEREST STATEMENT

The authors of the enclosed manuscript have no relevant financial or non‐financial interests to disclose.

## FUNDING INFORMATION

Not applicable.

## ETHICAL APPROVAL

Not applicable.

## Supporting information

Supporting Information

## References

[1] Rastogi K , Bhaskar S , Gupta S , Jain S , Singh D , Kumar P . Palliation of Brain Metastases: Analysis of Prognostic Factors Affecting Overall Survival. Indian J Palliat Care. 2018;24:308‐312. doi:10.4103/ijpc.ijpc_1_18 30111944 PMC6069611

[2] Sawaya R , Ligon BL , Bindal RK . Management of metastatic brain tumors. J Intensive Care Med. 1994;1:169‐178. 10.1007/BF02303562 7834443

[3] McTyre E , Scott J , Chinnaiyan P . Whole brain radiotherapy for brain metastasis. BMJ. 2013;4:S236‐S244. doi:10.4103/2152-7806.111301 PMC3656558 23717795

[4] Minniti G , Clarke E , Lanzetta G , et al. Stereotactic radiosurgery for brain metastases: analysis of outcome and risk of brain radionecrosis. Radiat Oncol. 2011;6:48. doi:10.1186/1748-717x-6-48 21575163 PMC3108308

[5] Patchell RA , Tibbs PA , Regine WF , et al. Postoperative radiotherapy in the treatment of single metastases to the brain: a randomized trial. JAMA. 1998;280:1485‐1489. 9809728 10.1001/jama.280.17.1485

[6] Cairncross JG , Kim JH , Posner JB . Radiation therapy for brain metastases. Cancer Radiother. 1980;7:529‐541. 10.1002/ana.410070606 7436358

[7] Hagen NA , Cirrincione C , Thaler HT , DeAngelis LM . The role of radiation therapy following resection of single brain metastasis from melanoma. Neurology. 1990;40:158‐160. 2296364 10.1212/wnl.40.1.158

[8] Aoyama H , Tago M , Kato N , et al. Neurocognitive function of patients with brain metastasis who received either whole brain radiotherapy plus stereotactic radiosurgery or radiosurgery alone. Int J Radiat Oncol Biol Phys. 2007;68:1388‐1395. 17674975 10.1016/j.ijrobp.2007.03.048

[9] Li J , Bentzen SM , Renschler M , Mehta MP . Regression after whole‐brain radiation therapy for brain metastases correlates with survival and improved neurocognitive function. J Clin Oncol. 2007;25:1260‐1266. 17401015 10.1200/JCO.2006.09.2536

[10] Mahajan A , Ahmed S , McAleer MF , et al. Post‐operative stereotactic radiosurgery versus observation for completely resected brain metastases: a single‐centre, randomised, controlled, phase 3 trial. Lancet Oncol. 2017;18(8):1040‐1048. doi:10.1016/s1470-2045(17)30414-x 28687375 PMC5560102

[11] Clarke JW , Register S , McGregor JM , et al. Stereotactic radiosurgery with or without whole brain radiotherapy for patients with a single radioresistant brain metastasis. Am J Clin Oncol. 2010;33:70‐74. doi:10.1097/coc.0b013e31819ccc8c 19652578

[12] Brown PD , Ballman KV , Cerhan JH , et al. Postoperative stereotactic radiosurgery compared with whole brain radiotherapy for resected metastatic brain disease (NCCTG N107C/CEC·3): a multicentre, randomised, controlled, phase 3 trial. Lancet Oncol. 2017;18:1049‐1060. doi:10.1016/s1470-2045(17)30441-2 28687377 PMC5568757

[13] Horsman MR , Overgaard J . The impact of hypoxia and its modification of the outcome of radiotherapy. J Radiat Res. 2016;57(Suppl 1):i90–i98. doi:10.1093/jrr/rrw007 26983987 PMC4990104

[14] Karlovits BJ , Quigley MR , Karlovits SM , et al. Stereotactic radiosurgery boost to the resection bed for oligometastatic brain disease: challenging the tradition of adjuvant whole‐brain radiotherapy. Neurosurg Focus. 2009;27(6):E7. doi:10.3171/2009.9.focus09191 19951060

[15] O'Brien DAR , Kaye SM , Poppas PJ , et al. Time to administration of stereotactic radiosurgery to the cavity after surgery for brain metastases: a real‐world analysis. J Neurosurg. 2021;135(6):1695‐1705. doi:10.3171/2020.10.jns201934 34049277

[16] Page MJ , McKenzie JE , Bossuyt PM , et al. The PRISMA 2020 statement: an updated guideline for reporting systematic reviews. BMJ. 2021;372:n71. doi:10.1136/bmj.n71 33782057 PMC8005924

[17] Gaspar L , Scott C , Rotman M , et al. Recursive partitioning analysis (RPA) of prognostic factors in three Radiation Therapy Oncology Group (RTOG) brain metastases trials. Int J Radiat Oncol Biol Phys. 1997;37:745‐751. doi:10.1016/s0360-3016(96)00619-0 9128946

[18] Sander C , Frydrychowicz C , Prasse G , et al. The impact of neurological performance and volumetrics on overall survival in brain metastasis in colorectal cancer: a retrospective single‐center case series. Bmc Cancer. 2022;22(1):336. doi:10.1186/s12885-022-09435-1 35346108 PMC8961891

[19] Sperduto PW , Kased N , Roberge D , et al. Summary report on the graded prognostic assessment: an accurate and facile diagnosis‐specific tool to estimate survival for patients with brain metastases. J Clin Oncol. 2012;30:419‐425. doi:10.1200/jco.2011.38.0527 22203767 PMC3269967

[20] McGrath S , Zhao X , Qin ZZ , Steele R , Benedetti A . One‐sample aggregate data meta‐analysis of medians. Stat Med. 2019;38:969‐984. doi:10.1002/sim.8013 30460713

[21] Won YK , Lee JY , Kang YN , et al. Stereotactic radiosurgery for brain metastasis in non‐small cell lung cancer. Radiat Oncol J. 2015;33:207‐216. doi:10.3857/roj.2015.33.3.207 26484304 PMC4607574

[22] Kasper E , Ippen F , Wong E , Uhlmann E , Floyd S , Mahadevan A . Stereotactic radiosurgery for brain metastasis from gynecological malignancies. Oncol Lett. 2017;13:1525‐1528. doi:10.3892/ol.2017.5621 28454285 PMC5403471

[23] Minniti G , Niyazi M , Andratschke N , et al. Current status and recent advances in resection cavity irradiation of brain metastases. Radiat Oncol. 2021;16:73. doi:10.1186/s13014-021-01802-9 33858474 PMC8051036

[24] Iorio‐Morin C , Masson‐Côté L , Ezahr Y , Blanchard J , Ebacher A , Mathieu D . Early Gamma Knife stereotactic radiosurgery to the tumor bed of resected brain metastasis for improved local control. J Neurosurg. 2014;121(Suppl):69‐74. doi:10.3171/2014.7.gks141488 25434939

[25] Choi JW , Im YS , Kong DS , Seol HJ , Nam DH , Lee JI . Effectiveness of Postoperative Gamma Knife Radiosurgery to the Tumor Bed After Resection of Brain Metastases. World Neurosurg. 2015;84(6):1752‐1757. doi:10.1016/j.wneu.2015.07.045 26211856

[26] Dasenbrock HH , Liu KX , Devine CA , et al. Length of hospital stay after craniotomy for tumor: a National Surgical Quality Improvement Program analysis. Neurosurg Focus. 2015;39:E12. doi:10.3171/2015.10.focus15386 26621410

[27] Kępka L , Tyc‐Szczepaniak D , Bujko K , et al. Stereotactic radiotherapy of the tumor bed compared to whole brain radiotherapy after surgery of single brain metastasis: Results from a randomized trial. Radiother Oncol. 2016;121(2):217‐224. doi:10.1016/j.radonc.2016.10.005 27793446

[28] Jagannathan J , Yen CP , Ray DK , et al. Gamma Knife radiosurgery to the surgical cavity following resection of brain metastases: Clinical article. J Neurosurg. 2009;111(3):431‐438. doi:10.3171/2008.11.jns08818 19361267

[29] Minniti G , Esposito V , Clarke E , et al. Multidose Stereotactic Radiosurgery (9 Gy × 3) of the Postoperative Resection Cavity for Treatment of Large Brain Metastases. Int J Radiat Oncol Biology Phys. 2013;86(4):623‐629. doi:10.1016/j.ijrobp.2013.03.037 23683828

[30] Hwang SW , Abozed MM , Hale A , et al. Adjuvant Gamma Knife radiosurgery following surgical resection of brain metastases: a 9‐year retrospective cohort study. J Neuro‐oncol. 2010;98(1):77‐82. doi:10.1007/s11060-009-0051-x 19904495

[31] Brennan C , Yang TJ , Hilden P , et al. A Phase 2 Trial of Stereotactic Radiosurgery Boost After Surgical Resection for Brain Metastases. Int J Radiat Oncol Biology Phys. 2014;88(1):130‐136. doi:10.1016/j.ijrobp.2013.09.051 PMC5736310 24331659

[32] Kalani MYS , Filippidis AS , Kalani MA , et al. Gamma Knife surgery combined with resection for treatment of a single brain metastasis: preliminary results: Clinical article. J Neurosurg. 2010;113(Special_Supplement):90‐96. doi:10.3171/2010.8.gks101067 21121791

[33] Iwai Y , Kazuhiro Y , Toshihiro Y . Boost radiosurgery for treatment of brain metastases after surgical resections. Surg Neurol. 2008;69(2):181‐186. doi:10.1016/j.surneu.2007.07.008 18261647

[34] Park YH , Park MJ , Ji SH , et al. Trastuzumab treatment improves brain metastasis outcomes through control and durable prolongation of systemic extracranial disease in HER2‐overexpressing breast cancer patients. Br J Cancer. 2009;100:894‐900. doi:10.1038/sj.bjc.6604941 19240719 PMC2661774

[35] Ushio Y , Arita N , Hayakawa T , et al. Chemotherapy of Brain Metastases from Lung Carcinoma: A Controlled Randomized Study. Neurosurgery. 1991;28(2):201‐205. doi:10.1227/00006123-199102000-00005 1997887

[36] Donovan EK , Parpia S , Greenspoon JN . Incidence of radionecrosis in single‐fraction radiosurgery compared with fractionated radiotherapy in the treatment of brain metastasis. Curr Oncol. 2019;26:e328‐e333. doi:10.3747/co.26.4749 31285676 PMC6588068

[37] Schüttrumpf LH , Niyazi M , Nachbichler SB , et al. Prognostic factors for survival and radiation necrosis after stereotactic radiosurgery alone or in combination with whole brain radiation therapy for 1–3 cerebral metastases. Radiat Oncol. 2014;9:105. doi:10.1186/1748-717x-9-105 24885624 PMC4036428

[38] Blonigen BJ , Steinmetz RD , Levin L , Lamba MA , Warnick RE , Breneman JC . Irradiated volume as a predictor of brain radionecrosis after linear accelerator stereotactic radiosurgery. Int J Radiat Oncol Biol Phys. 2010;77:996‐1001. doi:10.1016/j.ijrobp.2009.06.006 19783374

[39] Shaw E , Scott C , Souhami L , et al. Single dose radiosurgical treatment of recurrent previously irradiated primary brain tumors and brain metastases: final report of RTOG protocol 90‐05. Int J Radiat Oncol Biol Phys. 2000;47:291‐298. 10802351 10.1016/s0360-3016(99)00507-6

[40] Aoyama H , Shirato H , Tago M , et al. Stereotactic radiosurgery plus whole‐brain radiation therapy vs stereotactic radiosurgery alone for treatment of brain metastases: a randomized controlled trial. JAMA. 2006;295:2483‐2491. 16757720 10.1001/jama.295.21.2483

[41] Rabin BM , Meyer JR , Berlin JW , Marymount MH , Palka PS , Russell EJ . Radiation‐induced changes in the central nervous system and head and neck. Radiographics. 1996;16:1055‐1072. 8888390 10.1148/radiographics.16.5.8888390

[42] Shah R , Vattoth S , Jacob R , et al. Radiation necrosis in the brain: imaging features and differentiation from tumor recurrence. Radiographics. 2012;32:1343‐1359. doi:10.1148/rg.325125002 22977022

[43] Patel KR , Prabhu RS , Kandula S , et al. Intracranial control and radiographic changes with adjuvant radiation therapy for resected brain metastases: whole brain radiotherapy versus stereotactic radiosurgery alone. J Neuro‐oncol. 2014;120(3):657‐663. doi:10.1007/s11060-014-1601-4 25189789

[44] Patel RA , Lock D , Helenowski IB , et al. Postsurgical Cavity Evolution After Brain Metastasis Resection: How Soon Should Postoperative Radiosurgery Follow? World Neurosurg. 2018;110:e310‐e314. doi:10.1016/j.wneu.2017.10.159 29122731

