# Supplementary material for: Is there an optimal time to administer postoperative stereotactic radiosurgery in patients with brain metastases? A systematic review of the literature and meta‐analysis
Source: Precis Radiat Oncol. 2023 Dec 3;7(4):278–85. doi: 10.1002/pro6.1214 (PMC11935157; doi:10.1002/pro6.1214)
Supplement: Supplementary file 1 — Supporting Information [file PRO6-7-278-s001.docx]

SUPPLEMENTAL DATA

| **YEAR** | **AUTHOR** | **PATIENTS (N)** | **MEDIAN AGE (YEARS)** | **SEX (FEMALE)(%)** | **LOW RISK^1^ (%)** | **INTERMED RISK^2^ (%)** | **HIGH RISK^3^ (%)** | **GTR (%)** | **INFRATENT-ORIAL (%)** | **MEDIAN DOSE (GY)** | **MEDIAN TIMING TO pSRS (DAYS)** | **MEDIAN TREATMENT CAVITY VOLUME (ML)** | **LOCAL FAILURE (%)** | **REGIONAL FAILURE (%)** | **SYMPTOMATIC RADIONECROSIS (%)** | **RADIONECROSIS DIAGNOSIS METHOD** | **MEDIAN OS (MO.)** |
| --- | --- | --- | --- | --- | --- | --- | --- | --- | --- | --- | --- | --- | --- | --- | --- | --- | --- |
| 2019 | Dincoglan et al[1] | 28 | 55.5 | 52.0 | 46.5 | 46.5 | 7 | 100.0 | 43.0 | NR | NR | 27.2 | 25.0 | NR | 0.0 | MRI | 15.0 |
| 2017 | Mahajan et al. [2] | 63 | 58 | 52.0 | 60.0 | 29.0 | 11 | NR | NR | 16 | NR | 8.9 | 24.0 | 27.3 | 0.0 | MRI | 16.0 |
| 2016 | Shi et al. [3] | 29 | 59.5 | 52.0 | 69.0 | 21.0 | 7 | 83.0 | NR | NR | NR | NR | 26.0 | NR | 0.0 | PET-CT or MRS | NR |
| 2015 | Choi et al. [4] | 24 | 57 | 52.0 | 16.6 | 70.8 | 12.5 | NR | NR | 15 | 14.5 | 10.5 | 24.0 | NR | 0.0 | MRI | 11.0 |
| 2014 | Brennan et al. [5] | 49 | 59 | RN | 24.0 | 76.0 | 0 | 92.0 | 8.0 | 18 | 31.0 | NR | 15.0 | 44.0 | 17.5 | Histology | 14.7 |
| 2008 | Iwai et al. [6] | 21 | NR | 38.0 | NR | NR | NR | 86.0 | 43.0 | 17 | NR | NR | 24.0 | 48.0 | 0.0 | MRI | 20.0 |
| 2009 | Jagannathan et al. [7] | 47 | NR | RN | NR | NR | NR | 100.0 | NR | 19 | NR | NR | 6.0 | 72.0 | 0.0 | MRI | 10.0 |
| 2010 | Hwang et al. [8] | 25 | NR | 76.0 | NR | NR | NR | 95.0 | NR | NR | 28.0 | NR | 0.0 | 28.0 | 0.0 | MRI | 15.0 |
| 2009 | Karlovits et al. [9] | 52 | 61 | 46.1 | 42.3 | 57.6 | 0 | NR | 32.7 | 15 | 41.0 | 3.9 | 7.7 | 44.0 | 0.0 | MRI | 15.0 |
| 2010 | Kalani et al. [10] | 68 | NR | 54.4 | NR | NR | NR | 100.0 | 10.7 | 15 | 15.5 | 10.4 | 20.6 | NR | NR | MRI | 13.2 |
| 2011 | Jensen et al. [11] | 106 | 56.1 | 48.1 | NR | NR | NR | 96.4 | 27.7 | 17 | 24.0 | 8.0 | 14.2 | 53.7 | 2.8 | MRI | 10.9 |
| 2022 | Yaghi et al. [12] | 176 | 62 | 45.5 | NR | NR | NR | 90.9 | NR | 30 | 35.0 | NR | NR | 31.2 | NR | MRI | 9.8 |
| 2013 | Atalar et al. [13] | 63 | 59 | 70.0 | NR | NR | NR | 94.0 | NR | 18 | 16.0 | 14.5 | 10.3 | NR | NR | MRI, Metabolic imaging, Histology | 17.0 |
| 2018 | Patel et al. [14] | 79 | 61 | 52.0 | NR | NR | NR | 100.0 | 20.0 | NR | 20.0 | 11.5 | NR | NR | NR | NR | NR |
| 2019 | El Shafie et al. [15] | 24 | 60 | 66.6 | NR | NR | NR | NR | 33.0 | NR | 40.0 | 7.0 | 10.5 | 21.0 | 10.5 | MRI | NR |
| 2014 | Patel et al. [16] | 96 | 56 | 56.0 | NR | NR | NR | 74.0 | NR | 18 | 30.0 | 7.2 | 15.0 | 50.0 | 27.0 | MRI | NR |
| 2012 | Prabhu et al. [17] | 62 | 55 | NR | 24.0 | 68.0 | 15 | 81.0 | NR | 18 | 31.5 | 8.5 | 17.0 | 31.0 | 8.7 | Histology, PET-MRI, MRP, | 13.4 |
| 2012 | Robbins et al. [18] | 85 | 58 | 52.0 | NR | NR | NR | 68.0 | NR | 16 | 18.0 | NR | 18.8 | 55.0 | 8.0 | MRI, CT perfusion | 12.1 |
| 2012 | Steinmann et al. [19] | 33 | 58 | 58.0 | NR | NR | NR | NR | 15.0 | NR | 34.0 | 17.4 | 24.0 | 39.0 | 0.0 | MRI | 20.2 |
| 2013 | Broemme et al. [20] | 42 | 67 | 45.2 | NR | NR | NR | 83.0 | NR | NR | 40.0 | NR | 10.0 | 61.0 | 2.3 | Histology | 15.9 |
| 2013 | Minniti et al. [21] | 101 | 57 | 52.5 | 26.0 | 63.0 | 11 | NR | NR | 9 | 21.0 | 17.5 | 9.0 | 53.4 | 9.0 | MRI, Histology | 17.0 |
| 2014 | Ahmed et al. [22] | 65 | 61 | 49.2 | NR | NR | NR | 96.9 | NR | NR | 34.0 | 8.1 | 3.1 | 35.4 | 1.5 | MRI | 10.1 |

**Supplemental Fig 1.** Table illustrating the patient demographics, tumor characteristics, radiotherapy treatment regimen, and clinical outcomes for each publication. *Abbreviations*: *CT (computerized tomography), GPA (graded prognostic assessment), GTR (gross total resection), Gy (gray), intermed (intermediate), mL (milliliters), MO (months), MRC (medical research council), MRI (magnetic resonance imaging), MRP (magnetic resonance perfusion). MRS (magnetic resonance spectroscopy), NR (not reported), OS (overall survival), PET (positron emission tomography), pSRS (postoperative SRS), RPA (recursive partitioning analysis), SRS (stereotactic radiosurgery).*

^1^defined as RPA class I, GPA 1-2, MRC scale 1

^2^defined as RPA class II, GPA 2-3, MRC scale 2

^3^defined as RPA class III, GPA 3-4, MRC scale 3

| **STUDY METHODOLOGY** | **NUMBER OF ARTICLES** |
| --- | --- |
| Recorded adverse events related to SRS (non-radionecrosis) | 4 |
| Recorded adverse events related to surgical intervention | 3 |
| Radionecrosis confirmed with histology | 5 |
| Radionecrosis confirmed with PET scan, perfusion studies, MRS | 4 |

**Supplemental Fig 2.** Table detailing the number of articles using additional imaging or histological techniques in conjunction with MRI to diagnose radionecrosis, and articles that document non-radionecrosis adverse events related to surgical resection or SRS. *Abbreviations: MRI (magnetic resonance imaging), SRS (stereotactic radiosurgery).*


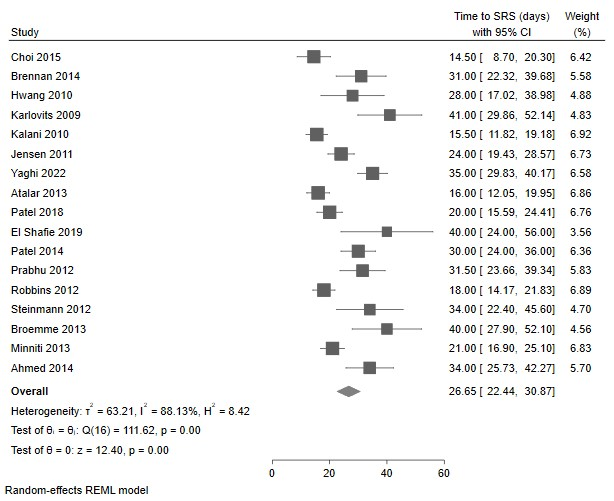


**Supplemental Fig. 3.** Random effects model demonstrating heterogeneity for time to SRS among the articles included in the meta-analysis (p<0.0001)


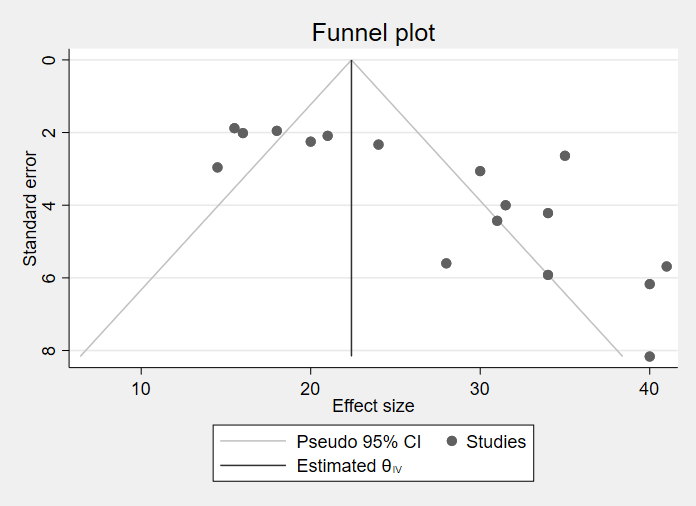


**Supplemental Fig. 4** Egger’s test used to evaluate for publication bias among the studies (p<0.0001)

1. Dincoglan F, Sager O, Uysal B, et al. Evaluatıon of hypofractıonated stereotactıc radıotherapy (HFSRT) to the resectıon cavıty after surgıcal resectıon of braın metastases: A sıngle center experıence. Indian J Cancer 2019; 56:202.

2. Mahajan A, Ahmed S, McAleer MF, et al. Post-operative stereotactic radiosurgery versus observation for completely      resected brain metastases: a single-centre, randomised, controlled, phase 3      trial. Lancet Oncol 2017; 18:1040–1048.

3. Shi S, Sandhu N, Jin MC, et al. Stereotactic Radiosurgery for Resected Brain Metastases: Single-Institutional Experience of Over 500 Cavities. Int J Radiat Oncol Biology Phys 2020; 106:764–771.

4. Choi JW, Im Y-S, Kong D-S, Seol HJ, Nam D-H, Lee J-I. Effectiveness of Postoperative Gamma Knife Radiosurgery to the Tumor Bed After      Resection of Brain Metastases. World Neurosurg 2015; 84:1752–7.

5. Brennan C, Yang TJ, Hilden P, et al. A Phase 2 Trial of Stereotactic Radiosurgery Boost After Surgical Resection for Brain Metastases. Int J Radiat Oncol Biology Phys 2014; 88:130–136.

6. Iwai Y, Yamanaka Kazuhiro, Yasui Toshihiro. Boost radiosurgery for treatment of brain metastases after surgical resections. Surg Neurol 2008; 69:181–186.

7. Jagannathan J, Yen C-P, Ray DK, et al. Gamma Knife radiosurgery to the surgical cavity following resection of brain metastases: Clinical article. J Neurosurg 2009; 111:431–438.

8. Hwang SW, Abozed MM, Hale A, et al. Adjuvant Gamma Knife radiosurgery following surgical resection of brain metastases: a 9-year retrospective cohort study. J Neuro-oncol 2010; 98:77–82.

9. Karlovits BJ, Quigley MR, Karlovits SM, et al. Stereotactic radiosurgery boost to the resection bed for oligometastatic brain      disease: challenging the tradition of adjuvant whole-brain radiotherapy. Neurosurg Focus 2009; 27:E7.

10. Kalani MYS, Filippidis AS, Kalani MA, et al. Gamma Knife surgery combined with resection for treatment of a single brain metastasis: preliminary results: Clinical article. J Neurosurg 2010; 113:90–96.

11. Jensen CA, Chan MD, McCoy TP, et al. Cavity-directed radiosurgery as adjuvant therapy after resection of a brain metastasis: Clinical article. J Neurosurg 2011; 114:1585–1591.

12. Yaghi NK, Radu S, Nugent JG, et al. Optimal timing of radiotherapy following brain metastases surgery. 2022; 9:133–141.

13. Atalar B, Choi CYH, Harsh GR 4th, et al. Cavity volume dynamics after resection of brain metastases and timing of      postresection cavity stereotactic radiosurgery. Neurosurgery 2013; 72:180–5; discussion 185.

14. Patel RA, Lock D, Helenowski IB, et al. Postsurgical Cavity Evolution After Brain Metastasis Resection: How Soon Should Postoperative Radiosurgery Follow? World Neurosurg 2018; 110:e310–e314.

15. Shafie RAE, Tonndorf-Martini E, Schmitt D, et al. Pre-Operative Versus Post-Operative Radiosurgery of Brain Metastases-Volumetric      and Dosimetric Impact of Treatment Sequence and Margin Concept. Cancers (Basel) 2019; 11.

16. Patel KR, Prabhu RS, Kandula S, et al. Intracranial control and radiographic changes with adjuvant radiation therapy for resected brain metastases: whole brain radiotherapy versus stereotactic radiosurgery alone. J Neuro-oncol 2014; 120:657–663.

17. Prabhu R, Shu H-K, Hadjipanayis C, et al. Current dosing paradigm for stereotactic radiosurgery alone after surgical      resection of brain metastases needs to be optimized for improved local control. Int J Radiat Oncol Biol Phys 2012; 83:e61-6.

18. Robbins JR, Ryu S, Kalkanis S, et al. Radiosurgery to the surgical cavity as adjuvant therapy for resected brain      metastasis. Neurosurgery 2012; 71:937–43.

19. Steinmann D, Maertens B, Janssen S, et al. Hypofractionated stereotactic radiotherapy (hfSRT) after tumour resection of a      single brain metastasis: report of a single-centre individualized treatment      approach. J Cancer Res Clin Oncol 2012; 138:1523–9.

20. Broemme J, Abu-Isa J, Kottke R, et al. Adjuvant therapy after resection of brain metastases. Frameless image-guided      LINAC-based radiosurgery and stereotactic hypofractionated radiotherapy. Strahlenther Onkol 2013; 189:765–70.

21. Minniti G, Esposito V, Clarke E, et al. Multidose Stereotactic Radiosurgery (9 Gy × 3) of the Postoperative Resection Cavity for Treatment of Large Brain Metastases. Int J Radiat Oncol Biology Phys 2013; 86:623–629.

22. Ahmed KA, Freilich JM, Abuodeh Y, et al. Fractionated stereotactic radiotherapy to the post-operative cavity for      radioresistant and radiosensitive brain metastases. J Neurooncol 2014; 118:179–86.
